# Novel Orthodontic Cement Comprising Unique Imidazolium-Based Polymerizable Antibacterial Monomers

**DOI:** 10.3390/jfb11040075

**Published:** 2020-10-17

**Authors:** Hui Lu, Xiaoming Jin

**Affiliations:** Dentsply Sirona, R&D—Consumables Product Group, 38 W Clarke Ave, Milford, DE 19963, USA

**Keywords:** white spot lesions, antibacterial, biofilms, dental, orthodontic

## Abstract

White spot lesions (WSLs) can develop quickly and compromise the successful outcome of the orthodontic treatment. Orthodontic bonding cement with the capability to prevent or mitigate WSLs could be beneficial, especially for patients with high risk of caries. This study explored novel mono- and di-imidazolium-based polymerizable antibacterial monomers and evaluated orthodontic cement compositions comprising such novel monomers. Their antibacterial potentials, mechanical properties, and shear bond strength (SBS) to bovine enamel were investigated. Statistical tests were applied to SBS and mechanical tests (one-way ANOVA and Tukey’s test). For antibacterial resins C (ABR-C) and E (ABR-E), their minimum inhibitory concentration (MIC) and minimum bactericidal concentration (MBC) against cariogenic *Streptococcus mutans* bacterial strain UA159 were found to be 4 μg/mL and 8 μg /mL, respectively. The loss of dry mass from completely demineralized dentin beams in buffer solutions pre-dipped into ABR-C and ABR-E resins is much less than that in control buffer (artificial saliva) only. For unfilled resins comprising up to 12 wt % ABR-C, no significant decreases in flexural strength or modulus were observed. For experimental cements incorporating 1–4 wt % ABR-C, there was no drastic compromise to the SBS to enamel except for 3 wt % ABR-C. Furthermore, their SBS was all comparable to the commercially available orthodontic cements. The ISO-22196 antimicrobial test against *S. aureus* showed significant levels of antibacterial effects—up to over 5 logs of microorganism reduction exhibited by ABR-C-containing experimental cements. The imidazolium-based polymerizable monomers could be utilized to functionalize orthodontic bonding cement with steady antibacterial activity and develop a potential strategy to counteract WSLs.

## 1. Introduction

Beyond just straightening teeth and achieving better esthetics, proper orthodontic intervention can result in greatly improved oral and psychological health [1,2]. A major undesirable outcome during orthodontic treatment is the development of white spot lesions (WSLs) around orthodontic brackets, especially near the gingival margin [3,4,5,6]. The boosted accumulation of biofilms around the brackets further lowers pH around these sites. Despite advancements in patient education, WSLs can still develop rapidly and could compromise the successful outcome of the treatment, even resulting in premature termination of treatment in severe cases [7,8,9]. It has been reported that between roughly 26% and 70% of patients using fixed orthodontic appliances exhibited various levels of WSLs during the orthodontic treatment [4,6,7]. In another study, the prevalence of WSLs was reported to be 38% in the 6-month group, whereas it was 46% in the 12-month group [9].

Due to the more retentive, complicated surfaces around brackets and higher occurrences for plaque or biofilm adherence, it has become quite challenging to maintain good oral hygiene, especially for less compliant patient groups [8,10,11,12]. It has been reported that fixed orthodontic appliances can affect the self-cleansing capabilities of the intraoral system, owing to the interactions of saliva and teeth surfaces. Even for patients with clear aligners, wearing aligners 20~22 h daily could limit the natural cleansing and neutralizing effects of saliva [13]. The fixed orthodontic appliances can even alter the oral microflora and increase the levels of acidogenic plaque bacteria, i.e., *Streptococcus mutans* (*S. mutans*), and *lactobacilli* in saliva and dental biofilm during active wearing of such appliances throughout lengthy orthodontic treatment procedures, with an average duration around two to three years [14,15,16,17,18,19] . 

The interface between tooth and orthodontic bonding adhesive or cement, many of them based on (meth)acrylate chemistry, could also become the victim of bacterial attack and subsequent degradation [20,21]. Finer et al.’s findings revealed increased degradation of resin-based composite and bonding agent by *S. mutans* UA159 vs. control. Esterase activities at levels that could degrade adhesives and resin composite have also been linked to *S. mutans* [20]. While bonding the irregularly shaped orthodontic appliances onto the tooth surface, the residual excess of orthodontic cements can also stimulate the extra accumulation of dental biofilm around brackets. The biofilm degradation of this important tooth–cement–bracket interface may contribute to the premature debonding of the brackets, especially given the compounding interactions with the microorganisms that could lead to biocorrosion on the surfaces of metal-based brackets and appliances [22].

Given the extensive prevalence of WSLs occurring during orthodontic treatments, various strategies have been utilized to prevent or mediate demineralization and occurrence of WSL formation. The use of fluoride via various forms, i.e., mouth rinse, gel, topical varnish, toothpaste, etc., has been the predominant remedy applied and studied [7,14,23,24]. Fluoride ions have been well documented to enhance remineralization of enamel and mitigate mineral loss during acid dissolution, by replacing the hydroxyl groups in hydroxyapatite and forming more acid-resistant fluorapatite [25]. The findings consolidated by Bergstrand and Twetman concluded that the use of topical fluoride varnish along with fluoride toothpaste has been the best evidence-based approach to prevent WSLs. The mean prevented portion based on six clinical trials was 42.5% with a range from 4% to 73% [23]. These studies provided convincing support for routine professional applications of fluoride varnish around the bracket base during orthodontic treatment [24,25,26,27,28].

The application of quaternary ammonium compound (QAC) has been actively investigated to introduce antibacterial activity into the orthodontic bonding system. A unique type of antimicrobial polymer is demonstrated by incorporating quaternary ammonium polyethyleneimine (QPEI) nanoparticles. QPEI nanoparticle-containing composites have been reported to exhibit antibacterial activity against salivary bacteria and in vivo antibiofilm activity [29]. Experimental orthodontic bonding cements with 1% incorporated insoluble QPEI and polycationic polyethyleneimine nanoparticles were reported to exhibit stable, long-lasting antibacterial properties against *S. mutans* [30,31]. A variety of QAC-containing functional monomers have been systematically studied, including the widely studied methacryloyloxydodecylpyridinium bromide (MDPB) [32,33] ; dimethylaminohexadecyl methacrylate (DMAHDM), a mono-methacrylate QAC with an alkyl chain length of 16 [34,35]; dimethylaminododedecyl methacrylate (DMADDM), a mono-methacrylate QAC with an alkyl chain length of 12 [36,37,38]; and 2-methacryloxylethyl hexadecyl methyl ammonium bromide (MAE-HB), a crosslinkable, di-methacrylate QAC with an alkyl chain length of 16 [39,40]. Significant antibacterial activities and biofilm reductions have been demonstrated.

Recently, a series of polymerizable imidazolium-based antibacterial resins (ABRs) were successfully designed in our laboratory [41,42]. To the best of our knowledge, no such novel crosslinkable imidazolium-based monomers have been investigated for their application towards an orthodontic bonding system with the potential to prevent and mitigate WSLs. The aims of this study were to investigate imidazolium-based ABR’s antibacterial activities and impacts on the adhesive and mechanical properties of experimental orthodontic bonding cement formulations.

## 2. Results

**Scheme 1:**
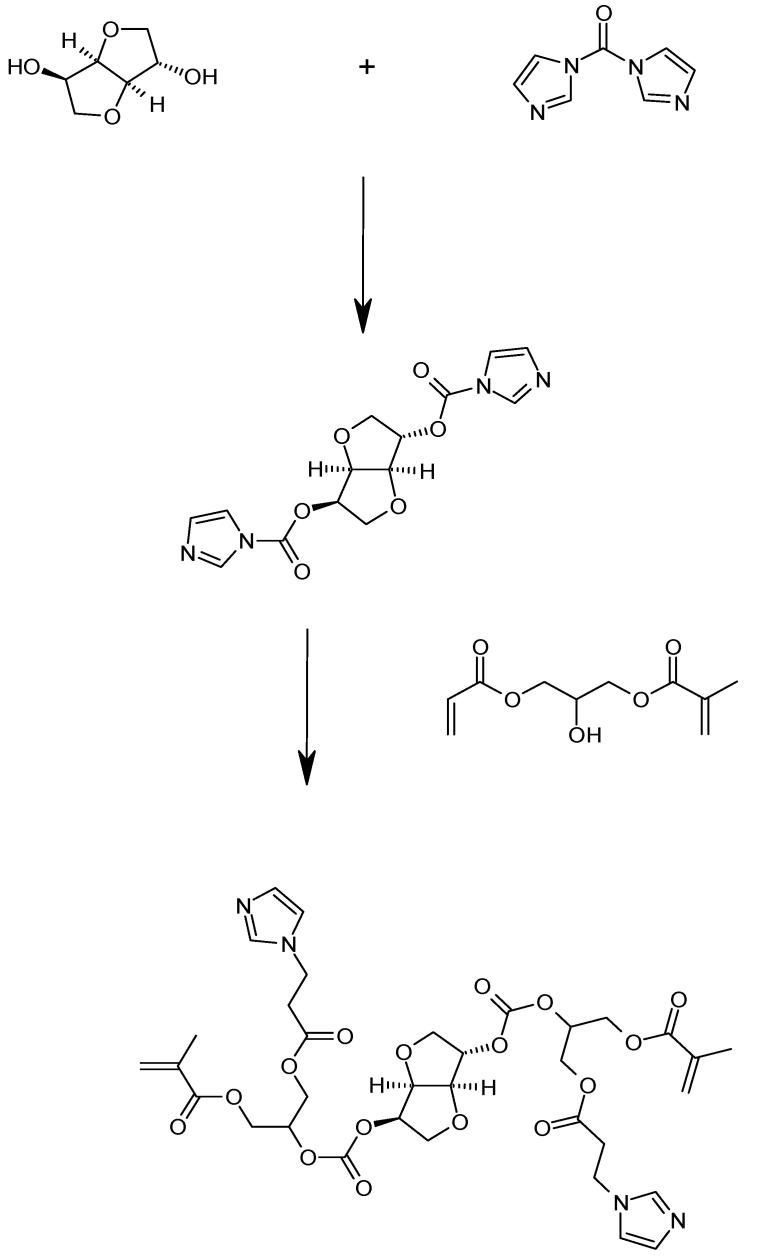
Isosorbide-based bisimidazole–dimethacrylate resins.

**Scheme 2:**
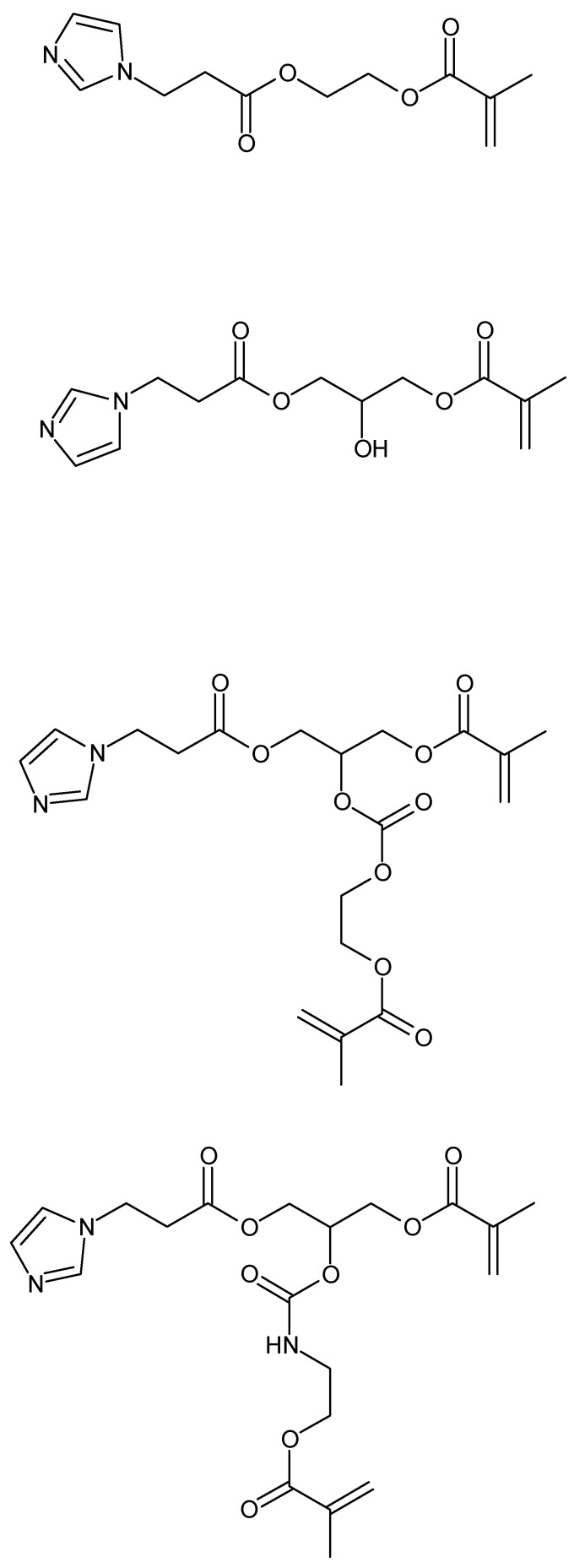
Various polymerizable mono-imidazole monomers synthesized.

By coupling with different mono-ols, diols, polyols (such as triols), monoamines, diamines, and/or polyamines, a range of methacrylate resins with (poly)imidazole moieties were prepared. During the processes of synthesizing imidazolium-based polymerizable resins from imidazole-containing monomers, a facile process based on imidazole and (meth)acrylated resins was developed. As demonstrated in Scheme 1 and Scheme 2, a variety of imidazole-containing polymerizable monomers were able to be prepared accordingly.

**Scheme 3:**
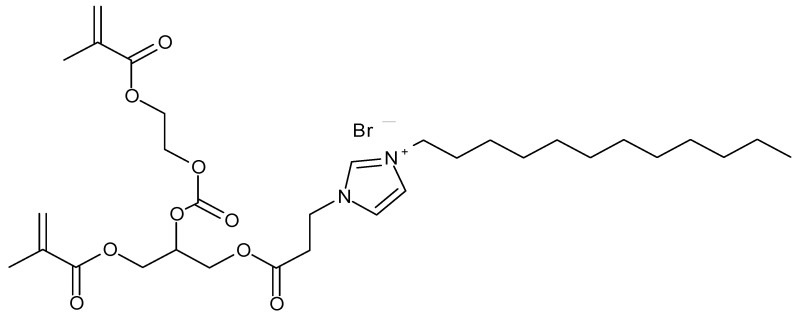
Polymerizable alkylimidazoliumbromide–dimethacrylate monomer, antibacterial resin C (ABR-C).

**Scheme 4:**
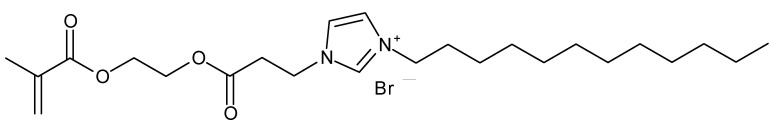
Polymerizable alkylimidazoliumbromide–methacrylate monomer ABR-E.

The polymerizable imidazole-based resins were further converted into polymerizable imidazolium-based monomers by reacting them with a variety of halogenated alkyls. A variety of polymerizable imidazolium-based antibacterial resins (ABRs) were successfully synthesized as exhibited in Scheme 3 and Scheme 4. The primary evaluations of these novel ABRs’ antibacterial activity include the determination of minimum inhibitory concentration (MIC) and minimum bactericidal concentration (MBC) against cariogenic *S. mutans* bacterial strain UA159. 

Table 1 presents the results of MIC and MBC for representative ABR resins and SDR Resin (a proprietary dimethacrylate monomer, without imidazole moiety, synthesized by Dentsply Sirona) in comparison to frequently used controls, namely chlorhexidine (CHX) as wide-spectrum antimicrobial agent and triethylene glycol dimethacrylate (TEGDMA). TEGDMA was chosen as control monomer because it is widely used in the composition of resins for dental-related applications, including but not limited to restorative, adhesive, and orthodontic compositions. In addition, it was not known to have antibacterial potencies against common cariogenic oral bacteria strains. Furthermore, by using a modified JIS Z 2801 test method, a preliminary antibacterial test against *S. mutans* strain UA159 was used to evaluate the antibacterial effectiveness for such formulated compositions. After 24 h contact, a bacterial reduction of 99.88% was exhibited by test sample containing ABR-E (Scheme 4), as compared to control. This indicated that imidazolium-based polymerizable resin could be highly effective in suppressing the growth of bacteria such as *S. mutans*.

**Figure 1:**
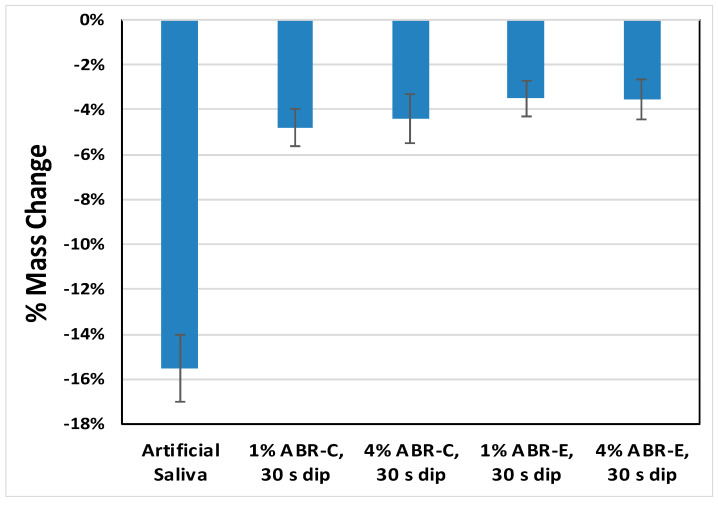
Effect of pre-dipping in antibacterial resins ABR-C and ABR-E on loss of dry mass of demineralized dentin beams after incubation for 7 days at 37 °C.

The loss of dry mass from completely demineralized dentin beams (2 mm × 1 mm × 6 mm) in buffer solutions pre-dipped into ABR-C and ABR-E resins, versus in control buffer (artificial saliva) without pre-dip, was studied. The amount of soluble collagen in these demineralized dentin beams was measured after 7 days of incubation at 37 °C. As Figure 1 indicates, dentin beams without pre-dip lost about 15.5% of their dry mass due to degradations of endogenous matrix metalloproteinases (MMPs) and cathepsins. When the dentin beams were pre-dipped in 1–4% of imidazolium-based ABR resins (ABR-C, ABR-E) for 30 s and then dropped in incubation medium, the dentin beams only lost about 3.5–5% of their dry mass. Furthermore, the hydroxyproline (HYP), an amino acid unique to collagen, content in the medium was also measured. The collagen peptide fragments that were solubilized by the endogenous proteases of the dentin matrix were hydrolyzed to amino acids in 6N HCl and then analyzed for hydroxyproline. In the control group, the proteases released about 10.5 µg HYP/mg dry dentin after 7 days; beams pre-dipped in 1% or 4% of ABR-C released less than half as much hydroxyproline and the beam treated with ABR-E yielded a similar result.

As the designed imidazolium-based resin still comprises a methacrylate functional group, no compatibility or copolymerization issues were found when mixed with typical dimethacrylate-type dental resins, such as 1,6-bis[methacryloyloxyethoxycarbonylamino]-2,4,4-trimethylhexane (UDMA), ethoxylated bisphenol A dimethacrylate (EBPADMA), and TEGDMA. Even with 16 wt % loading of ABR-C into the control orthodontic resin, no mixability issue was observed and the resulting resin mixture was found uniform. The 3-point bending flexural strength and modulus of unfilled orthodontic resins containing 4~16 wt% ABR-C, along with control, were measured following ISO-4049 method and the results are presented in Table 2.

**Figure 2:**
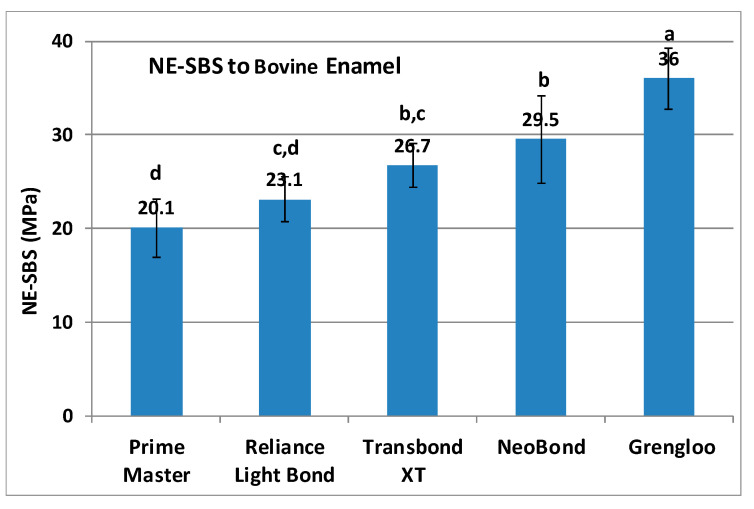
Notched-edge shear bond strength to bovine enamel of commercial orthodontic cements. Values that do not share the same superscript(s) are significantly different (α = 0.05).

**Figure 3:**
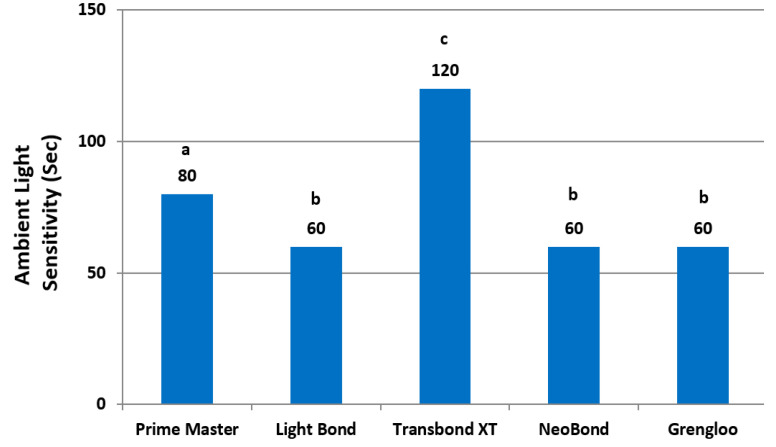
Ambient light sensitivity of commercial orthodontic cements. Values that do not share the same superscript are significantly different (α = 0.05).

The 75 wt % filled experimental orthodontic cements incorporating various concentrations of antibacterial monomer ABR-C were also studied. The current investigation examined flexural and compressive strengths, flexural modulus, ambient light sensitivity, and notched-edge shear bond strength (NE-SBS) to bovine enamel. Results along with statistical analysis are presented in Table 3. For reference, the shear bond strength to bovine enamel, using the same NE-SBS method, and ambient light sensitivity of five commercially available orthodontic bonding cements were also evaluated, as presented in Figure 2 and Figure 3, respectively.

Antibacterial testing was also conducted at an independent and good laboratory practice (GLP) complied testing institution. As shown in Table 4, for orthodontic cement paste formulations that incorporated imidazolium-based dimethacrylate antibacterial monomer (ABR-C), the ISO-22196 antimicrobial testing results against ATCC 6538 showed significant levels of antibacterial effects—up to over 5 logs of microorganism reduction—when compared with control. Such highly effective bactericidal effects for the imidazolium-based polymerizable resins were promising due to a relatively low-level loading and significantly reduced probability of leaching out owing to the dimethacrylate functional groups.

## 3. Discussion

As demonstrated by MIC and MBC test results reported in Table 1, ABR-C and ABR-E displayed distinct antibacterial activities against cariogenic *S. mutans* cells. Not surprisingly, the MIC values of ABR-C and ABR-E were higher than that of benchmark wide-spectrum bactericidal chlorhexidine, which is also highly leachable and does not offer sustained antibacterial activity once leached out. Also as expected, the 8 µg/mL MBC of ABR-C and ABR-E is higher than CHX’s MBC of 4 µg/mL, whereas no bactericidal activities were observed at all for SDR Resin and TEGDMA. It has been shown that, for an organic QAS compound, 10~14 carbon atoms for the N-alkyl group are associated with higher antimicrobial activity [43,44]. Another study also showed that imidazolium’s N-alkyl’s chain length can play a critical role in antitumor activity and cytotoxicity; C-12 was found exhibiting high antitumor activity against ~60 tumor cell lines as well as low cytotoxicity in most cases. Longer chain length could improve antitumor potency but also increase imidazolium’s cytotoxicity [45]. As demonstrated in their chemical structures in Scheme 3 and Scheme 4, respectively, both ABR-C and ABR-E have a 12-carbon atom chain length on their N-alkyl group, which helps in explaining their identical MIC and MBC values.

The dentin mass loss results (Figure 1) indicate that the ABR resins, even at low concentrations, are effective against matrix metalloproteinases (MMPs) for minimizing the collagen degradation. MMPs belong to a larger group of proteases known as the metzincin superfamily. These collagenases are capable of degrading triple-helical fibrillary collagens, such as those in dentin, into distinctive fragments. More specifically, certain MMPs are considered to contribute to the gradual degradation of collagen fibrils in hybrid layers established during dentin bonding. A wide range of MMPs, including MMP-2, MMP-8, MMP-9, and MMP-20, have been detected in the human dentin matrix [46]. It is well reported that CHX has broad anti-MMP activity in addition to antimicrobial capability. However, the long-term *in vivo* anti-MMP activity of CHX may not be as effective, which has been attributed to possible leaching out of the CHX [47,48]. It has also been reported that cationic quaternary ammonium methacrylates may exhibit dentin MMP inhibition comparable with that of CHX but that higher concentrations were required [49].

The synthetic strategy in this investigation focused on covalently linking antibacterial compounds that contain functional groups with strong antibacterial activities to a variety of backbones and polymerizable groups compatible with conventional dental resin systems. The antibacterial capability of the active compound could be either enhanced or reduced by polymerization. This depends on how the compound eradicates bacteria, either by disrupting bacterial membrane or via depleting the bacterial food supply. In order for an antibacterial resin to be a viable option for industrial-scale production and application, there are several essential requirements that need to be fulfilled: (1) synthesis of ABR should be straightforward and not cost-prohibitive; ideally, well-established synthetic routes are preferred; (2) ABR should have good storage stability (sufficient shelf life); (3) end products that contain ABR should be able to maintain their antibacterial activities through recurrent bacterial challenges; (4) ideally, the antibacterial effect can be achieved with relatively small dosing and minimum bacterial resistance in the target pathogenic microorganisms; (5) no drastic compromises to the other vital properties should be introduced by ABR. As shown in Table 2, even at up to 12 wt % loading level, there are no significant decreases in flexural strength or flexural modulus with the incorporation of the imidazole-based polymerizable antibacterial monomer ABR-C, as compared to control. The flexural strength of the resin mixture with 16 wt % antibacterial monomer showed lower flexural strength, but flexural modulus still retained 84% value as compared to control.

During this investigation, it was found that a group of polymethacrylate resins containing at least one imidazole moiety could be readily prepared via appropriate hybrid methacrylate–acrylate resins or polyacrylate resins with proper control of the conversion of the imidazole addition. This is an effective approach to incorporating an imidazole moiety into a polymerizable resin as novel acid-free functional resins. Moreover, such imidazole-containing polymerizable resins may be further chemically modified by reacting with a variety of halogenated alkyls to form polymerizable resins with ionic moiety of imidazolium, which results in a unique class of polymerizable ionic liquid resins. 

Sufficient mechanical properties of the orthodontic bonding cement are important as the bending force and torque during teeth straightening and aligning could be quite significant. There is a slight reduction of flexural strength with a higher loading amount of ABR-C; however, flexural modulus can be maintained with up to 3 wt % of ABR-C loading (Table 3). A similar trend was also observed for compressive strength. Ambient light sensitivity (ALS) is another important property for light curable orthodontic cement or adhesive, as adequate ALS is needed for the bracket to be carefully placed and aligned at the correct position and angle, before it is light cured. As also exhibited in Table 3, the ALS values of ABR-C-containing orthodontic cements are all at 2 min or higher, which compared well with commercially available orthodontic cements, as shown in Figure 3.

Orthodontic cement with the capability to prevent the development of WSLs without compromising enamel bond strength would be desirable. Orthodontic treatments last an average of two to three years, during which time orthodontic cement must be capable of bonding to the enamel with high bond strength in order to resist masticatory loads. As demonstrated in Table 3, for experimental orthodontic cements incorporating 1–4 wt % ABR-C, there is no drastic compromise to the SBS to enamel except for 3 wt % ABR-C. More importantly, their SBS values are all comparable to those of the commercially available orthodontic cements products (Figure 2), with mean SBS ranging from 20.1 MPa to 36.0 MPa. However, this bond strength to enamel should not be too high either, to prevent damages to the enamel while removing the bracket.

The strong effectiveness in eradicating bacteria for the imidazolium-based polymerizable resins was further demonstrated by the formulated experimental orthodontic cements. As exhibited in Table 4, even at a low-level loading (1 wt %) of such imidazolium-based polymerizable monomer, a reduction of over 2 logs of microorganisms can be achieved by ABR-C. When the loading level of ABR-C was increased from 2 wt % to 4 wt %, reductions of around 5 logs or higher were observed. Moreover, with optimized compositions, not only can a highly effective antibacterial capability be achieved, but balanced mechanical properties can also be maintained (Table 3). The potent antibacterial property of this novel polymerizable imidazolium resin offers another crucial benefit—no severe cytotoxicity was introduced [50]. Conventional QAS-based polymerizable resins, on the other hand, could be less effective and a high dose loading (up to 30 wt %) is needed, which frequently leads to significant drops in mechanical properties and elevated cytotoxicity [51,52].

Effective antimicrobial agents are expected to regulate the oral biofilm at levels compatible with good oral health while not sacrificing the beneficial properties of the resident oral microflora. Compounds comprising imidazolium moieties have exhibited various antibacterial, antioxidant, and antifungal properties; imidazolium salts’ overall mechanism of antibacterial function is similar to that of QAS—mainly by disturbing the planktonic cell membrane [52]. Nonetheless, comprehensive studies by Koo et al. have shown that ABR-MC (ABR-modified composite) impaired biofilm initiation by disrupting bacterial accumulation, cell colonization, and subsequent biofilm development, if left unintervened [50]. The anti-biofilm properties of ABR-MC were assessed using an EPS-matrix producing *S. mutans* in an experimental biofilm model. Using high-resolution confocal fluorescence imaging and biophysical methods, they observed severely reduced biomass, remarkable disruption of bacterial accumulation, and defective 3D matrix structure on the surface of ABR-MC. Mechanism-wise, it was speculated that the distinct difference in the basicity between N-substitute imidazole (pK_aH_ of 7.20 for 1-methylimidazole) and aliphatic tertiary amine (pK_aH_ of 10.7 for triethylamine) might be the major contributor to the increased polarity and higher potential in its charged conjugated moiety (imidazolium). However, the exact mechanism contributing to the much more pronounced bactericidal effects as compared to conventional QAS compounds will require further investigation. Other areas of relevant investigation include the further study of the hydrolytical stability of these antibacterial monomers.

## 4. Materials and Methods

### 4.1. Materials

Antibacterial resins (ABR-C and ABR-E) were prepared in house; the detailed preparation route can be found in a previous paper [50]. SDR Resin and adhesion-promoting resin (APR, Dentsply proprietary resin) were supplied by Dentsply Sirona’s internal production (Milford, DE, USA). Other conventional dental resins and ingredients used in formulations were purchased from commercial sources, including 1,6-bis [methacryloyloxyethoxycarbonylamino]-2,4,4-trimethylhexane (UDMA), ethoxylated bisphenol A dimethacrylate (EBPADMA), trimethylolpropane trimethacrylate (TMPTMA), camphorquinone (CQ), dimethylaminobenzonitrile (DMABN), diphenyl (2,4,6-trimethylbenzoyl) phosphine oxide (TPO), and butylated hydroxytoluene (BHT). Inorganic fillers were milled and silanated in house, including barium fluoroalumino borosilicate glass (BFBG), bariumalumino borosilicate glass (BABG), and AEROSIL fumed silica OX-50 (silanated OX-50). All fillers were surface treated by γ-methacryloxypropyl-trimethoxysilane.

Commercially available orthodontic cements studied include the following: Prime Master (Sun Medical Co., Shiga, Japan), Light Bond (Reliance Orthodontic Products, Alsip, IL, USA), Transbond XT (3M Unitek, Monrovia, CA, USA), NeoBond (Dentsply Sirona, Milford, DE, USA), and Grengloo (Ormco, Glendora, CA, USA).

Preparation of experimental orthodontic resin and cement. Experimental dental resin mixture with various concentrations of polymerizable antibacterial resin (0 (control), 4, 8, 12, 16 wt %) was mixed with other resins, namely UDMA (28~34 wt %), EBPADMA (20~26 wt %), APR (28~32 wt %), TMPTMA (6~8 wt %), CQ (~0.1 wt %), DMABN (~0.2 wt %), and BHT (~0.07 wt %), to a uniform resin blend at 55 °C using an overhead mechanical stirrer (Fisher Scientific, Pittsburgh, PA, USA). Experimental orthodontic cements were compounded with 75 wt % of inorganic filler mixture using a Ross double planetary mixer at 55 °C to a uniform paste (Ross Mixer, Charles Ross & Son Company, Hauppauge, NY, USA). The inorganic filler used in this study consists of three types of fillers in the filler blend: ~33 wt % BABG, ~65 wt % BFBG-2, and ~2 wt % silanated OX-50.

### 4.2. Evaluation Methods

MIC and MBC Testing. Three monomers were tested against controls, i.e., ABR-E, ABR-C, and SDR Resin (a proprietary dimethacrylate monomer, without imidazole moiety, synthesized by Dentsply Sirona). The monomers were diluted in UltraPure distilled water (Gibco, Gaithersburg, MD, USA) before use. Different concentrations of the three tested monomers, along with two controls, i.e., wide-spectrum antimicrobial agent chlorhexidine (CHX) and conventional monomer TEGDMA, were incubated statically with the cariogenic organism *S. mutans* strain UA159 using mid-exponential cells for 48 h at 37 °C in the presence of 5% CO_2_. A fixed initial inoculum size of 9 × 10^7^ CFU/mL of bacterial cells was inoculated per well and 2-fold dilutions of the experimental monomers, CHX, and TEGDMA were added. The following concentrations were tested: 0 to 125 µg/mL for CHX; 0 to 0.1% for the experimental monomers ABR-E, ABR-C, SDR Resin, and the control monomer TEGDMA. The MIC was determined as the lowest test concentration needed to ensure that culture did not grow over 10% of the relative cell, as determined by visual inspection of the growth inhibition of each well compared to that of the control well (without test compounds). For the MBC test, cells were serially diluted and spot-plated on agar plates; MBC values were determined by using the actual reduction of the bacterial viable cell counts. The MBC was determined as the lowest concentration that allows less than 0.1% of the original inoculum to survive.

Dentin Mass Loss. Completely demineralized dentin beams (2 mm × 1 mm × 6 mm) were prepared. Dentin beams were obtained from extracted molars after removing the enamel and superficial dentin. The beams were then submerged in 10% phosphoric acid for 18 h at 25 °C to completely demineralize the dentin. They were incubated in a control buffer (artificial saliva) to see how much of the insoluble collagen in demineralized dentin could be solubilized in 7 days of incubation at 37 °C. The loss of dry mass from completely demineralized dentin beams (2 mm × 1 mm × 6 mm) in buffer solutions pre-dipped into ABR-C and ABR-E resins for 30 s, versus in control buffer (artificial saliva) without pre-dip, was studied. The amount of soluble collagen in these demineralized dentin beams was measured after 7 days of incubation at 37 °C.

Flexural Strength. Specimens were prepared based on ISO 4049:2019. Samples were carefully injected into 25 mm × 2 mm × 2 mm stainless steel molds, then covered by Mylar film and cured with a Spectrum 800^®^ (Dentsply Sirona, Milford, DE, USA ) halogen lamp uniformly across the entire length of the specimen, using an irradiance of 850 mW/cm^2^ for 5 × 20 s. After curing, specimens were immersed in DI water for 24 h at 37 °C prior to the 3-pt bending flexural test. Flexural test was performed on an Instron Universal Tester (Model 4400R, Norwood, MA, USA countr using a crosshead speed of 0.75 mm/min. Six specimens per sample set were prepared and tested.

Compressive Strength. Samples were carefully injected into ∅4 mm × 7 mm stainless steel molds, sandwiched between two Mylar films. Light curing of the specimen was carried out with a Spectrum 800^®^ (Dentsply Sirona) halogen lamp at an irradiance of 850 mW/cm^2^ for 20 s on both ends. After curing, specimens were immersed in DI water for 24 h at 37 °C, before being polished to ∅4 mm × 6 mm specimen size with 600 grit sand paper. The compression test was performed on an Instron Universal Tester (Model 4400R) using a crosshead speed of 5 mm/min. Six specimens per sample set were prepared and tested.

Ambient Light Sensitivity (ALS). ALS was tested on a Suntest unit (model CPS 56007014, Heraeus Noblelight, Buford, GA, USA ) with a UV filter that was calibrated to provide illuminance of 8000 ± 1000 lux tested by a Lux Meter HD400 (Extech, Nashua, NH, USA ). A spheroidal mass of approximately 0.03 gm of the material was weighed to be tested on a glass microscope slide, and the slide was positioned with a matte black cover on top of the cell with a UV filter in place. The Suntest light was turned on, and one should wait at least 10 s until the light output has stabilized. The matte black cover was removed and the timer was started simultaneously. After exposure, the slide with the sample was removed and the second microscope slide was immediately pressed against the material with a slight shearing action to produce a thin layer. The material was visually inspected to see whether it was physically homogeneous. If the material has begun to set, clefts and voids appear in the specimen when the thin layer is being produced. The time was recorded when the sample started to set and appeared physically non-homogeneous. In all, 3 runs were conducted for each sample.

Notched-Edge Shear Bond Strength. Caries-free and freshly extracted bovine incisors were carefully sectioned longitudinally through the distal, occlusal, and mesial surfaces using a precision low speed saw (TechCut 4™, Allied High Tech Products, Rancho Dominguez, CA, USA). The sectioned bovine incisors, with buccal surface exposed, were then mounted in a cylindrical block using cold-cure acrylics. The exposed surface was then coarse ground on a model trimmer until a flat enamel surface was exposed, followed by wet ground on a grinding wheel under running water using 120-grit and 320-grit SiC sanding papers until the surface was even and smooth when visually inspected. Enamel was acid etched using 34% phosphoric etching gel for 15 s, then thoroughly rinsed for 20 s. The notched-edge bonding jig contained a cylindrical plastic mold, resulting in samples with a defined bonding area (diameter of 2.38 mm). The antibacterial orthodontic cement restorative composite was then carefully placed into the center of the mold and good contact with the substrate was ensured. After light curing with a Spectrum 800^®^ (Dentsply Sirona) halogen lamp at 850 mW/cm^2^ for 20 s, the specimen was then carefully removed from the mold. Specimens were stored in DI water for 24 h at 37 °C prior to SBS testing. The SBS test was performed on an Instron Universal Tester (Model 4400R) using a crosshead speed of 1 mm/min. Seven specimens per sample set were prepared and tested.

JIS Z 2801. This test was conducted at Antimicrobial Test Laboratories (ATL, Round Rock, TX, USA), an independent and GLP complied testing institution. JIS Z 2801 was adopted as ISO standard (ISO-22196). This test is designed to quantitatively test the ability of hard surfaces to inhibit the growth of microorganisms over a 24-h period of contact. The test microorganism selected for this test was *Streptococcus mutans* (*S mutans*, ATCC^®^ 25175). The following protocol was used by ATL: (1) test microorganism *S mutans*, ATCC^®^ 25175 was prepared by growth in tryptic soy broth for 24 h; (2) the suspension of the test microorganism was standardized by dilution in a Dey-Engley neutralizing broth (D/E Broth) at 0.9 mL blanks; (3) control and test surfaces were inoculated with microorganisms, and then the microbial inoculum was covered with a thin sterile film; (4) microbial concentrations were determined at “time zero” by elution followed by dilution and plating to tryptic soy agar (Difco); (5) a control was run to verify that the neutralization/elution method effectively neutralized the antimicrobial agent in the antimicrobial surface being tested; (6) the inoculated covered control and antimicrobial test surfaces were allowed to incubate undisturbed in a humid environment for 24 h at 36 ± 1 °C; and (7) after incubation, microbial concentrations were determined and the reduction of microorganisms relative to the control surface was calculated as follows:

R (Average Log Reduction) = Log (B/C),(1)

where

B = Average number of viable cells on the control pieces after 24 h.
C = Average number of viable cells on the test pieces after 24 h.

ISO-22196 Antimicrobial Test. This test was conducted at Antimicrobial Test Laboratories (ATL, Round Rock, TX, USA), an independent and GLP complied testing institution. ISO method 22196 is a quantitative test designed to assess the performance of materials’ antimicrobial capabilities on hard non-porous surfaces. The method can be conducted using contact times ranging from 10 minutes up to 24 h. For the ISO 22196 test, non-antimicrobial control surfaces are used as the baseline for calculations of microbial reduction. The test microorganism selected for this test was *Staphylococcus aureus* 6538 (*S. aureus* 6538). This bacterium is a Gram-positive, spherical-shaped, facultative anaerobe. *Staphylococcus* species are known to demonstrate resistance to antibiotics such as methicillin and is commonly used in standard test methods as a model for Gram-positive bacteria. *S. aureus* pathogenicity can range from commensal skin colonization to more severe diseases such as pneumonia and toxic shock syndrome (TSS). The following protocol was used by ATL: (1) *S. aureus* 6538 prepared by growth in tryptic soy broth for 18 h and the suspension of the test microorganism was standardized by dilution in broth at 1:500 by volume; (2) control and test surfaces were inoculated with microorganisms, and then the microbial inoculum was covered with a sterile thin film; (3) microbial concentrations were determined at “time zero” by elution, followed by dilution and plating to tryptic soy agar; (4) a control was run to verify that the neutralization/elution method effectively neutralized the antimicrobial agent in the antimicrobial surface being tested; (5) the inoculated covered control and test surfaces were allowed to incubate undisturbed in a humid environment at 36 ± 1 °C for 24 h; and (6) after incubation, microbial concentrations were determined and the reduction of microorganisms relative to the control surface was calculated as follows:

$$Percent\;Reduction=(\frac{B-A}{B})\times 100$$

$$Log_{10}\;Reduction=Log(\frac{B}{A})$$

where

B = Number of viable test microorganisms on the control carriers after the contact time.
A = Number of viable test microorganisms on the test carriers after the contact time.

Statistical Analysis. Statistical analysis was conducted with Minitab 17 Statistical Software (Minitab^®^, State College, PA, USA). Normality of data was checked using the Anderson–Darling test. Mechanical properties and shear bond strength data were analyzed with one-way analysis of variance (ANOVA) and Tukey’s test. Significance levels of 0.05 were used for ANOVA and Tukey’s tests.

## 5. Conclusions

This study explored the potential of incorporating unique imidazolium-based polymerizable antibacterial monomers into orthodontic bonding cements. For unfilled resins comprising up to 12 wt % ABR-C, no significant decreases in flexural strength or modulus were observed. For experimental cements incorporating 1–4 wt % ABR-C, there is no drastic compromise to the SBS to enamel except for 3 wt % ABR-C; moreover, their SBS values are all comparable to those of the commercially available orthodontic cements. The ISO-22196 antimicrobial test against *S. aureus* showed significant levels of antibacterial effects—up to over 5 logs of microorganism reduction exhibited by ABR-C-containing experimental cements. Although MIC and MBC were identified, and a range of antibacterial activities of imidazolium-based orthodontic cements were demonstrated, clinical and long-term studies are still important to validate the efficacy of this unique series of imidazolium resins towards preventing and mitigating WSLs for patients.

## Figures and Tables

**Table 1:** Minimum inhibitory concentration (MIC) and minimum bactericidal concentration (MBC) of novel antibacterial resins ABR-E and ABR-C against *Streptococcus mutans* strain UA159, as compared to SDR Resin, triethylene glycol dimethacrylate (TEGDMA), and chlorhexidine.

Test Compound	Stock Solution	Tested Concentrations	Bacterial Inoculum Size (CFU/mL)	MIC	MBC
ABR-E	2.5%	0–0.1%	9 × 10^7^	4 µg/mL (0.0004%)	8 µg/mL (0.0008%)
ABR-C	1%	0–0.1%	9 × 10^7^	4 µg/mL (0.0004%)	8 µg/mL (0.0008%)
SDR Resin	0.25%	0–0.1%	9 × 10^7^	No bacterial growth inhibition	
TEGDMA	100%	0–0.1%	9 × 10^7^	No bacterial growth inhibition	
Chlorhexidine	2.5 mg/mL	0–125 mg/mL	9 × 10^7^	2 mg/mL	4 mg/mL

**Table 2:** Flexural strength and flexural modulus of exp. orthodontic resins (unfilled) that contain various concentrations of imidazolium-based polymerizable antibacterial monomer ABR-C.

Resin Sample	Control	4%_ABR-C	8%_ABR-C	12%_ABR-C	16%_ABR-C
**Antibacterial Monomer in Resin**	0	4 wt%	8 wt%	12 wt%	16 wt%
**Flexural Strength, MPa (s.d.)**	97 (8) ^A^	88 (5) ^A,B^	91 (4) ^A^	95 (3) ^A^	81 (4) ^B^
**Flexural Modulus, MPa (s.d.)**	2443 (87) ^a^	2388 (183) ^a^	2394 (139) ^a^	2293 (82) ^a,b^	2125 (125) ^b^

Within the same row, means that do not share the same superscript(s) are significantly different (α = 0.05).

**Table 3:** Adhesive and physical properties of orthodontic cements (filled with 75% inorganic filler) that contain various concentrations of imidazolium-based polymerizable antibacterial monomer ABR-C.

Orthodontic Cement	Control	1%_ABR-C	2%_ABR-C	3%_ABR-C	4%_ABR-C
**Antibacterial Monomer in Cement**	**0**	**1 wt%**	**2 wt%**	**3 wt%**	**4 wt%**
**Resin Conc.**	25.0%	25.0%	25.0%	25.0%	25.0%
**Ambient Light Sensitivity**	2:35"	2:00"	2:05"	2:00"	2:15"
**Compressive Strength, MPa (s.d.)**	373 (25) ^A^	361 (12) ^A,B^	346 (10) ^B^	352 (5) ^A,B^	307 (15) ^C^
**Flexural Strength, MPa (s.d.)**	150 (14) ^A^	139 (6) ^A^	114 (10) ^B^	114 (10) ^B^	88 (7) ^C^
**Flexural Modulus, MPa (s.d.)**	11165 (531) ^a^	10848 (383) ^a,b^	10319 (549) ^a,b^	10607 (629) ^a,b^	10066 (445) ^b^
**NE-SBS to Etched Enamel, MPa (s.d.)**	32.8 (2.7) ^A,B^	36.7 (4.5) ^A^	28.8 (4.3) ^B,C^	25.4 (3.5) ^C^	31.0 (5.8) ^A,B,C^
**SBS to Enamel Range, MPa**	29.5 ~ 37.2	31.9 ~ 43.4	21.9 ~ 32.7	21.5 ~ 29.5	23.1 ~ 38.0
**SBS to Enamel C.V.**	8.1%	12.1%	14.8%	14.0%	18.7%

Within the same row, means that do not share the same superscript(s) are significantly different (α = 0.05).

**Table 4:** ISO-22196 antimicrobial test of orthodontic cements (filled with 75 wt % inorganic filler) that contain various concentrations of imidazolium-based polymerizable antibacterial monomer using *S. aureus* 6538. The limit of detection for this assay is 5 CFU/carrier.

Test Micro-Organism	Contact Time	Carrier Type	Anti-Bacterial Monomer in Cement	CFU/Carrier	Percent Reduction Compared to Control at Contact Time	Log_10_ Reduction Compared to Control at Contact Time
*S. aureus* 6538	Time Zero	Control	0	1.00 × 10^6^	N/A	
	24 h	ATL Control	0	8.50 × 10^5^		
		2%_ABR-C	2 wt%	1.00 × 10	99.9988%	4.93
		4%_ABR-C	4 wt%	1.50 × 10	99.998%	4.75
		1%_ABR-C	1 wt%	4.72 × 10^3^	99.44%	2.26
		3%_ABR-C	3 wt%	<5.00	> 99.9994%	> 5.23
